# Persisting symptoms in patients with Hashimoto’s disease despite normal thyroid hormone levels: Does thyroid autoimmunity play a role? A systematic review

**DOI:** 10.1016/j.jtauto.2021.100101

**Published:** 2021-04-15

**Authors:** Karelina L. Groenewegen, Christiaan F. Mooij, A.S. Paul van Trotsenburg

**Affiliations:** Department of Pediatric Endocrinology, Emma Children’s Hospital, Amsterdam UMC, University of Amsterdam, Amsterdam, the Netherlands

**Keywords:** Hashimoto’s disease, Persisting symptoms, Quality of life, Thyroid auto-immunity, Hypothyroidism

## Abstract

**Objective:**

Patients with hypothyroidism due to Hashimoto’s disease (HD) may experience persisting symptoms despite normal serum thyroid hormone (TH) levels. Several hypotheses have been postulated to explain these persisting symptoms. We hypothesized that thyroid autoimmunity may play a role.

**Design:**

A systematic literature review.

**Methods:**

A PubMed search was performed to find studies investigating the relation between the presence of thyroid autoimmunity and (persisting) symptoms. Included studies were critically appraised by the Newcastle – Ottawa Scale (NOS) and then subdivided into (A) disease-based studies, comparing biochemically euthyroid patients with HD, and euthyroid patients with non-autoimmune hypothyroidism or euthyroid benign goitre, and (B) (general) population-based studies. Due to different outcome measures among all studies, meta-analysis of data could not be performed.

**Results:**

Thirty out of 1259 articles found in the PubMed search were included in this systematic review. Five out of seven disease-based studies found an association between thyroid autoimmunity and symptoms or lower quality of life (QoL). Sixteen of 23 population-based studies found a comparable positive association. In total, the majority of included studies reported an association between thyroid autoimmunity and persisting symptoms or lower QoL in biochemically euthyroid patients.

**Conclusion:**

(Thyroid) autoimmunity seems to be associated with persisting symptoms or lower QoL in biochemically euthyroid HD patients. As outcome measures differed among the included studies, we propose the use of similar outcome measures in future studies. To prove causality, a necessary next step is to design and conduct intervention studies, for example immunomodulation vs. placebo preferably in the form of a randomized controlled trial, with symptoms and QoL as main outcomes.

## Highlights

• This review reports on a relation between thyroid autoimmunity and symptoms.
• Persisting symptoms in euthyroid HD patients may be associated with autoimmunity.
• Thyroid autoimmunity and symptoms were associated in population-based studies.

## 1 Introduction

Hypothyroidism is defined as lower than optimal thyroid hormone (TH) production by the thyroid gland, resulting in too low or suboptimal plasma TH concentrations [1]. The most frequent cause of hypothyroidism is thyroid dysfunction, also known as primary hypothyroidism [2]. This type of hypothyroidism is characterized by a low or (low-)normal serum free thyroxine (FT4) concentration in combination with a (very) high or elevated thyrotropin (TSH) concentration, and can be a congenital or acquired problem. Worldwide, the most frequent causes of acquired primary hypothyroidism are iodine deficiency and (chronic) autoimmune thyroiditis [3,4].

Autoimmune thyroiditis or Hashimoto’s disease (HD) is an autoimmune disorder, in which T- and B cells (slowly) destruct the thyroid gland. A key role in this process seems to be reserved for cytotoxic T cells that are activated by excessively stimulated CD4 positive T cells. Serological markers are anti-thyroid antibodies: anti-thyroid peroxidase and anti-thyroglobulin antibodies (TPO- and Tg-abs, respectively), produced by B cells [5,6].

In case of clinical suspicion of hypothyroidism, thyroid function assessment consists of TSH and FT4 measurement [6]. HD is diagnosed by the presence of TPO- or Tg-abs, plus or minus characteristic thyroid ultrasound abnormalities, such as reduced echogenicity [3,6,7]. Common, but also quite unspecific complaints in (primary) hypothyroidism are fatigue, weight gain with poor appetite, constipation, concentration problems and depression [[8], [9], [10]]. Treatment of hypothyroidism consists of daily administration of levothyroxine (LT4) [6,8,11]. LT4 is preferable to triiodothyronine (T3) because of its longer serum half-life [6,11]. Furthermore, the thyroid gland mainly produces thyroxine (T4), which is converted into its active metabolite T3 in peripheral tissues [6,8].

Despite normalized TSH and FT4 levels by LT4 treatment, approximately five to ten percent of HD patients experience persisting symptoms [[12], [13], [14]]. With respect to these persisting symptoms, several hypotheses have been postulated and discussed: 1) TSH is not a perfect marker; consequently, there standard LT4 treatment may not result in a truly biochemically euthyroid state [4]; therefore, some experts suggest to treat with a supraphysiological LT4 dose, which would result in a suppressed TSH, but fewer complaints; however, not all studies show the same results, and higher LT4 doses may increase the risk of cardiovascular disease; 2) a healthy thyroid gland produces approximately 80–90% T4 and 10–20% T3 [8,11,15]; since not all administered LT4 will be converted into active T3, combination therapy of LT4 and LT3 may result in less persisting symptoms; however, until now, it has not been shown that adding LT3 is better than LT4 alone [8]; 3) since deiodinase type 2 (DIO2) facilitates peripheral deiodination of T4 into active T3, patients with *DIO2* gene polymorphisms may have variable peripheral T3 availability; in such cases LT4 treatment alone may not be enough [16,17]; with the Thr92Ala *DIO2* polymorphism being present in 12–36% of the population [18], this might explain persisting symptoms in a considerable part of affected patients. Yet, none of these three hypotheses about the cause of persisting symptoms in treated patients with HD has been definitely proven. Therefore, according to the American Thyroid Association guideline from 2014, currently LT4-monotherapy is the best choice of treatment in hypothyroidism [8].

In the past years results of several studies have suggested that persisting symptoms in HD patients may be related to autoimmunity [[19], [20], [21]]; for example, in a systematic review Siegmann et al. reported a possible correlation between depression and anxiety disorders, and thyroid autoimmunity [22]. While hypothyroidism in HD patients is treated with TH, the autoimmune process affecting the thyroid gland is left untreated. Although, it has been shown that serum TPO-Ab levels decline in most patients with HD who are taking LT4 after a mean of 50 months, TPO-Ab levels became negative in only 16% of the studied patients, illustrating that the majority of patients have persisting elevated TPO-Ab levels [23]. We therefore hypothesized that persisting symptoms in treated patients with HD may be related to autoimmunity. Already in the 1960s [24], it has been recognized that, regardless of thyroid function, *thyroid* autoimmunity may *cause* neurological or psychiatric symptoms; in the absence of another obvious cause this clinical picture was called Hashimoto’s encephalopathy. The idea that *thyroid* autoimmunity causes the encephalitis has been abandoned, and is replaced by the hypothesis that these patients suffer from autoimmunity that not only affects the thyroid, but also the brain. Hence the name “Steroid-Responsive Encephalopathy with Autoimmune Thyroiditis” (SREAT). With this in mind, we hypothesized that persisting symptoms encountered in TH treated HD patients also results from autoimmunity affecting the brain. Besides *thyroid* autoimmunity other latent autoimmune diseases could hypothetically play a role in persisting symptoms in treated HD patients. A recent meta-analysis showed that (latent) poly-autoimmunity is common in patients with an autoimmune thyroid disorder. However, its effect on the course of the persisting symptoms is still unclear [25].

The main objective of this systematic review was to find out whether or not the presence of *thyroid* autoimmunity is associated with persisting symptoms in HD patients. We performed a literature search in PubMed for original studies investigating the relation between the presence of thyroid autoimmunity and symptoms performed in (LT4 treated) *euthyroid* patients with hypothyroidism due to HD compared with *euthyroid* patients with non-autoimmune hypothyroidism or *euthyroid* benign goitre screened for persisting symptoms, or in general or specific non-HD populations (persons positive or negative for anti-thyroid antibodies, screened for symptoms with specific questionnaires). The “general populations” consisted of either healthy persons, or of patients prone for autoimmune thyroid disease because of already existing other autoimmune disease.

## 2 Methods

This systematic review was performed following the Preferred Reporting Items for Systematic Reviews and Meta-Analysis (PRISMA) 2020 guidelines [26].

### 2.1 Information sources and literature search

For this systematic review the PubMed database was searched for relevant articles. The search was conducted with Mesh and TIAB key terms, using the components Population and Outcome of the PICO-strategy by Glasziou et al.: ‘autoimmune hypothyroidism’ and ‘persisting symptoms’, respectively [27]. The following equivalents of these key terms were used: 1) autoantibodies, autoimmunity, autoantigens, autoantibody, antibody, 2) hypothyroidism, 3) thyroglobulin, iodide peroxidase, thyrotropin receptors, TSH receptor, TPO, peroxidase, 4) Hashimoto disease, autoimmune thyroiditis, Hashimoto, Hashimoto’s thyroiditis, 5) brain diseases, behavioural symptoms, mental disorders, signs and symptoms, quality of life, brain, fatigue, depression. The equivalent terms were combined as follow: ((1 AND (2 OR 3)) OR 4) AND 5.

### 2.2 Study selection and quality assessment

Title and abstract screening were performed independently by two of the authors (KLG and ASPvT). Full text screening was performed together. Inclusion criteria were original studies investigating the relation between the presence of thyroid autoimmunity and symptoms performed in (LT4 treated) *euthyroid* patients with hypothyroidism due to HD compared with *euthyroid* patients with non-autoimmune hypothyroidism or *euthyroid* benign goitre who were screened for persisting symptoms, or in well-defined (general) populations. Exclusion criteria were: review articles, case reports or series, articles in other languages than English, articles about thyroid antibodies without any relation to thyroid disorders, and articles about HD in relation to other diseases than persisting symptoms or quality of life. Part of the full text screening was a critical appraisal to evaluate the quality of each study following the Newcastle-Ottawa Scale (NOS), a quality assessment form for cohort studies. In this eight-item checklist, we considered thyroid autoimmunity as the ‘exposure’, and - as explained earlier - the various persisting symptoms as the ‘outcome’. Due to the cross-sectional design of many of the included studies, these could not be fully scored on all aspects (e.g., follow-up) of the outcome domain. The original thresholds for good or fair studies were therefore not always applicable. Nevertheless, the NOS scale gave a good indication of the quality of the included studies.

As already mentioned in the introduction, included articles were categorized in either 1) disease-based studies: groups of (LT4 treated) *euthyroid* patients with hypothyroidism due to HD compared with *euthyroid* non-autoimmune hypothyroidism patients or *euthyroid* patients with benign goitre, or 2) (general) population-based studies. In the population-based studies the results of different well-being questionnaires were analysed in relation to the presence or absence of thyroid auto-antibodies in well-defined groups. These studies were subsequently subdivided into 2A) studies in healthy persons, and 2B) studies performed in patients prone for thyroid autoimmunity, and thus prone for poly-autoimmunity, because of already existing other autoimmune disease, e.g., rheumatoid arthritis or celiac disease. The main reasons for making this subdivision were that patients suffering from other autoimmune disease might have had knowledge of having a higher chance of also developing autoimmune thyroid disease, and that symptoms of “other” autoimmune diseases may resemble those of autoimmune thyroid disease/HD. This makes results from these studies related to the research question of this systematic review somewhat more difficult to interpret. Nonetheless, these studies were included if there was independent evaluation of a possible relation between thyroid autoimmunity and (persisting) symptoms or QoL.

### 2.3 Data presentation

Disease-based studies are presented according to the way patients were assessed or to used outcome measure, in the following order: 1) well-being (including all neuropsychological tests, quality of life, fatigue and other mood-parameters) and 2) brain-function (including functional imaging of the brain). Results of the population-based studies are presented in the following order: 1) (truly) healthy persons, 2) individuals recruited from primary care facilities, 3) postpartum women, 4) pregnant women, 5) perimenopausal women, 6) individuals with an already existing other autoimmune disease.

Data are presented as four evidence tables, two for the population-based studies, and two for the disease-based studies: study and patient characteristics (Table 1, Table 3), and study results (Table 2, Table 4)***.***

**Table 1:** Study and patient characteristics of disease-based studies.

Article	Research question	Study design	Patients			Controls		
			Sample size (N)	Gender F (N)	Mean Age in yrs (±SD, range)	Sample size (N)	Gender F (N)	Mean Age in yrs (±SD, range)
**STUDIES WITH OUTCOME MEASUREMENT: WELL-BEING**								
ZIVALJEVIC 2015^**26**^	What is the QoL of HT patients compared to patients with BG? And does thyroid surgery improve the health of this patients even with normal hormonal status on LT4 treatment?	Cohort study, prospective	27 euthyroid HT (LT4 treatment)	26 (96%)	52.2 (±10.9, median: 52.0)	116 euthyroid BG (LT4 treatment)	99 (85%)	52.6 (±12.9, median: 55.0)
GIYNAS AYHAN 2014^**27**^	What is the current prevalence of major depression and anxiety disorders in patients with euthyroid HT and euthyroid goiter? And does HT increases the risk of depressive or anxiety disorders compared with endemic/non-endemic goiter or controls?	Cohort study, retrospective	51 euthyroid HT (no treatment)	49 (96.1%)	35.1 (±7.75, 20-45)	45 euthyroid endemic / non-endemic goiter (no treatment)	41 (91.1%)	35.47 (±6.74)
LOUWERENS 2012^**28**^	What is the impact of the cause of hypothyroidism on fatigue and fatigue-related symptoms in patients treated for hypothyroidism of different origin (AIH vs. DTC)?	Cross-sectional study	138 euthyroid AIT (LT4 treatment)	119 (86.2%)	48.3 (±9.8)	140 euthyroid DTC (LT4 treatment)	114 (81.4%)	49.3 (±13.3)
BAZZICHI 2012^**29**^	Is there a predisposition for the development of FM in patients with HT with or without SCH compared with SCH alone?	Cross-sectional study	21 SCH + HT	-	-	13 SCH without HT	12 (92.3%)	38.54 (±15.33)
OTT 2011^**19**^	Are higher anti-TPO levels associated with an increased symptom load and a decreased QoL in a female euthyroid patient cohort? (with/without treatment)	Cohort study, prospective	47 Anti-TPO >121.0 IU/mL	47 (100%)	52.3 (±12.7)	379 Anti-TPO ≤121.0 IU/mL	379 (100%)	54.6 (±12.0)
**STUDIES WITH OUTCOME MEASUREMENT: BRAIN FUNCTION**								
LEYHE 2013^**21**^	Is there an association between the performance in *d2* attention testing and GM density of the LIFG on MRI in euthyroid HT patients compared to euthyroid patients undergoing hormonal substitution for goiter or after thyroid surgery?	Cohort study, retrospective	13 euthyroid HT (treatment)	11 (84.6%)	43.0 (±12)	12 euthyroid goiter/post-surgery (treatment)	9 (75.0%)	47.0 (±13)
LEYHE 2008^**20**^	Is there a neuropsychological impairment in a subgroup of HT patients indicating a subtle brain dysfunction independent of thyroid dysfunction?	Cohort study, prospective	26 euthyroid HT (treatment)	23 (88.5%)	46.0 (±1.9)	25 euthyroid goiter/post-surgery (treatment)	19 (82.6%)	49.8 (±1.9)

AIH ​= ​autoimmune hypothyroidism, AIT ​= ​autoimmune thyroiditis, anti-TPO ​= ​anti thyroid peroxidase, BG ​= ​benign goiter, DTC ​= ​differentiated thyroid carcinoma, FM ​= ​fibromyalgia, GM = grey matter, HT = Hashimoto’s thyroiditis, LIFG ​= ​left inferior frontal gyrus, LT4 ​= ​Levothyroxine 4, MRI ​= ​magnetic resonance imaging, QoL ​= ​quality of life, SCH ​= ​subclinical hypothyroidism.

**Table 2:** Results of disease-based studies.

ARTICLE	MAIN OUTCOME MEASURE	RESULTS			CONCLUSION	DO RESULTS SUPPORT THE HYPOTHESIS?
**STUDIES WITH OUTCOME MEASUREMENT: WELL-BEING**						
ZIVALJEVIC 2015^26^	Histologically confirmed HT or BG.	HT N=27	Other BG N=116	P-value	Preoperatively, hypothyroid symptoms were significantly more expressed; sex life was significantly worse in HT patients than in BG patients (p=0.025 and p=0.007, respectively). Pre-operative differences in other domains were not significant.	No, *except for a significantly worse QoL score on the domain “sex life”*
	QoL pre-operative in ThyPRO domains:				Postoperatively, goiter symptoms were significantly worse in the HT patients than in the BG patients (=0.042). This result is probably due to the fact that an inflammatory process develops around the thyroid gland in HT patients, but not in BG patients.	
	- Goiter symptoms	21.4 (±11.2)	20.9 (±14.0)	0.505		
	- Hyperthyroid symptoms	16.3 (±11.7)	20.3 (±14.7)	0.159		
	- Hypothyroid symptoms	24.8 (±22.1)	15.0 (±15.9)	0.025		
	- Eye symptoms	4.9 (±7.2)	6.2 (±11.0)	0.579		
	- Tiredness	33.6 (±20.2)	35.7 (±23.7)	0.957		
	- Cognitive impairment	13.4 (±20.0)	13.6 (±17.0)	0.926		
	- Anxiety	21.8 (±20.8)	23.1 (±18.9)	0.564		
	- Depression	27.6 (±16.3)	25.4 (±18.1)	0.377		
	- Emotional susceptibility	19.5 (±18.9)	22.5 (±17.7)	0.330		
	- Social life	9.0 (±18.9)	7.9 (±13.1)	0.579		
	- Daily life	16.3 (±14.2)	15.3 (±17.0)	0.289		
	- Sex life	20.0 (±24.7)	14.4 (±27.7)	0.007		
	- Cosmetic complaints	11.6 (±14.0)	10.9 (±13.7)	0.777		
	- Overall QoL	36.1 (±28.0)	33.0 (±28.8)	0.473		
	QoL post-operative in ThyPRO domains:					
	- Goiter symptoms	2.9 (±5.1)	1.8 (±6.6)	0.042		
	- Hyperthyroid symptoms	9.1 (±10.4)	8.3 (±7.4)	0.853		
	- Hypothyroid symptoms	15.0 (±15.9)	10.6 (±11.7)	0.176		
	- Eye symptoms	4.5 (±7.6)	2.1 (±4.5)	0.110		
	- Tiredness	20.5 (±14.1)	17.5 (±14.4)	0.125		
	- Cognitive impairment	8.9 (±11.4)	9.9 (±13.9)	0.948		
	- Anxiety	10.8 (±14.8)	10.6 (±16.8)	0.891		
	- Depression	15.7 (±13.1)	15.0 (±12.4)	0.834		
	- Emotional susceptibility	10.2 (±9.6)	9.5 (±9.0)	0.717		
	- Social life	5.3 (±10.9)	3.4 (±6.7)	0.537		
	- Daily life	6.2 (±8.9)	7.6 (±12.1)	0.803		
	- Sex life	4.2 (±11.5)	3.5 (±12.3)	0.556		
	- Cosmetic complaints	2.9 (±5.5)	1.5 (±4.2)	0.078		
	- Overall QoL	2.8 (±8.0)	3.4 (±11.8)	0.845		
GIYNAS AYHAN 2014^27^	Prevalence of psychiatric diagnoses according DSM-IV:	HT N =51	Goiter N=45	P-value	A higher percentage of major depression, OCD, PD, GAD, an anxiety disorder, any depressive disorder and any psychiatric disorder was found in the HT group compared with the goiter group. However, differences were not statistically significant.	No
	- Major depression	15 (29.4%)	10 (22.2%)	0.489		
	- Dysthymic disorder	2 (3.9%)	4 (8.9%)	0.414		
	- OCD	8 (15.7%)	3 (6.7%)	0.209		
	- PD	6 (11.8%)	3 (6.7%)	0.495		
	- GAD	3 (5.9%)	1 (2.2%)	0.620		
	- Phobic disorder	3 (3.9%)	3 (6.7%)	0.663		
	- An anxiety disorder	19 (37.3%)	11 (24.4%)	0.194		
	- Any depressive disorder	17 (33.3%)	11 (24.4%)	0.375		
	- Any psychiatric disorder	27 (52.9%)	17 (37.8%)	0.155		
LOUWERENS 2012^28^	Scores on five MFI-20 subscales between AIH and DTC:	AIH N=138	DTC N=140	P-value	Patients with AIH were significantly more fatigued in contrast to patients with hypothyroidism after total thyroidectomy, which could not be attributed to thyroid or clinical parameters. Therefore these findings probably represent a disease-specific decrease in QoL	Yes
	- General fatigue	15.1 ± 4.3	11.0 ± 4.8	<0.001		
	- Physical fatigue	13.0 ± 4.1	9.9 ± 4.9	<0.001		
	- Reduction in activity	11.6 ±4.6	8.8 ± 4.1	<0.001		
	- Reduction in motivation	11.0 ± 4.4	8.6 ± 3.8	<0.001		
	- Mental fatigue	12.7 ± 4.9	9.5 ± 4.8	<0.001		
BAZZICHI 2012^29^	FM comorbidity in HT patients with SCH compared to SCH alone. Scores of FIQ and VAS for fatigue and pain in the different studied group of patients.	HT + SCH N=21 28.5%	SCH alone N=13 0.0%	P-value	HT patients with FM comorbidity had a significantly higher mean duration of disease with respect to the all other thyroid patients (8.50 ±6.20 vs. 3.67 ±2.75 years, P=0.0022).	Yes
		N=39	N=13	-	HT patients (SCH+/-) had a higher incidence of clinical symptoms and significantly higher values of FIQ, VAS pain and VAS fatigue scores compared to patients affected by SCH alone.	
	- FIQ (mean, SD)	43.13 (24.97)	17.39 (14.48)	0.001		
	- VAS fatigue (mean, SD)	4.51 (3.36)	1.54 (2.54)	0.006		
	- VAS pain (mean, SD)	3.03 (3.19)	0.38 (0.77)	0.009		
OTT 2011^19^	Thyroid histology based calculation of anti-TPO concentration cut-off, predictive of lymphocytic infiltration of the thyroid gland.	Anti-TPO >121IU/mL N=47	Anti-TPO ≤121 IU/mL N=379	P-value	Histologically confirmed HT showed significantly higher anti-TPO levels than those without histological signs of HT.	Yes
	Preoperative general symptom questionnaire				Increased anti-TPO levels were found to be associated with a lower quality of life and various general symptoms (chronic fatigue, dry hair, getting easily fatigued, dysphagia, chronic irritability, chronic nervousness).	
	- Chronic fatigue	31 (66.0%)	185 (48.8%)	0.027		
	- Dry skin	24 (51.1%)	168 (44.3%)	0.381		
	- Dry hair	18 (38.3%)	77 (20.3%)	0.005		
	- Vaginal dryness	8 (17%)	58 (15.3%)	0.759		
	- Chronic sensation of cold	14 (29.8%)	82 (21.6%)	0.207		
	- Frequent sweating	23 (48.9%)	165 (43.5%)	0.482		
	- Becoming easily fatigued	21 (44.7%)	111 (29.3%)	0.031		
	- Chronic weakness	7 (14.9%)	39 (10.3%)	0.034		
	- Dysphagia	15 (31.9%)	63 (16.6%)	0.011		
	- Chronic weeping	13 (27.7%)	86 (22.7%)	0.447		
	- Chronic irritability	21 (44.7%)	95 (25.1%)	0.004		
	- Chronic lack of concentration	15 (31.9%)	71 (18.7%)	0.033		
	- Chronic nervousness	36 (67.6%)	149 (39.3%)	<0.001		
	- Frequent mood swings	17 (36.2%)	110 (29.0%)	0.312		
		Anti-TPO >121 IU/mL	Anti-TPO ≤121 IU/mL			
	QoL by SF-36 Questionnaire:	N=78	N=346			
	- General health	61.3±22.6	68.2±17.6	0.015		
	- Physical functioning	75.9±22.5	82.8±20.8	0.062		
	- Role physical	68.2±37.1	80.3±31.6	0.011		
	- Bodily pain	74.7±22.6	80.3±23.8	0.137		
	- Vitality	50.5±17.3	57.0±18.5	0.025		
	- Social functioning	74.4±21.6	82.3±20.8	0.020		
	- Role emotional	78.8±31.4	80.2±33.5	0.798		
	- Mental health	61.7±20.3	66.8±18.0	0.050		
	Correlation between histological signs of HT with anti-TPO levels:	HT 367.4±134.7 IU/ml	Non-HT 28.2±65.7 IU/ml	<0.001, *r*^2^ = 0.46		
**STUDIES WITH OUTCOME MEASUREMENT: BRAIN FUNCTION**						
LEYHE 2013^21^	Neurocognitive function assessed by the *d2* attention test.	No significant differences in the total score of the *d2* attention test were detected between groups (P=0.9).			Performance in attention testing is associated with GM density LIFG in patients with HT, but not in patients with other thyroid diseases. Particularly low achievement was associated with reduced GM density of this brain region suggesting an influence of autoimmune processes on the frontal cortex in this disease. This could be due to not yet known antibodies affecting brain morphology or an influence of thyroid antibodies themselves.	Yes
	GM density on MRI was correlated with *d2* test scores.	A significant correlation between GM density and *d2* test total score could be shown for the opercular part of the LIFG (MNI coordinates: *x*=038, *y*=25, Z score=3.89, *k*=153 voxels, *P*<0.05) in patients with HT (*r*=0.88, *P*<0.001, Cohen’s *d*=1.89), but not in the control group (*r*=-0.06, *P*=0.94, Cohen’s *d*=-0.06).				
		A negative relationship between GM density and level of TPO-Abs was found in the HT patients which, however, scarcely failed to reach significance (*r*=-0.44, *P*=0.06, Cohen’s *d*=0.49).				
LEYHE 2008^20^	Neurocognitive function assessed by the *d2* attention test.	Control group N=25	Hashimoto’s thyroiditis N=26	P-value	No significant differences between groups were detected comparing the main values of the performances in the neuropsychological tests. However, significantly more patients with HT than in controls were found with z-scores below the normal range (less than -1.5) in de d2 attention test regarding total scores.	Yes
	Number of patients below the normal range (z-scores).				These results point to subtle brain dysfunction in a group of patients with HT who were euthyroid and without diagnosed neuropsychiatric disease.	
	- D2 total score I: total number of items processed minus errors.	3	10	0.0302		
	- D2 total score II: number of correctly processed items minus errors.	1	11	0.0013		

AIH ​= ​autoimmune hypothyroidism, anti-TPO ​= ​anti-TPO ​= ​anti thyroid peroxidase, BG ​= ​benign goiter, DSM-IV ​= ​diagnostic and statistical manual of mental disorders IV, DTC ​= ​differentiated thyroid carcinoma, FM ​= ​fibromyalgia, FIQ ​= ​fibromyalgia impact questionnaire, GAD ​= ​generalized anxiety disorder, GM ​= ​grey matter, HT = Hashimoto’s thyroiditis, LIFG ​= ​left inferior frontal gyrus, MFI-20 ​= ​multidimensional fatigue inventory, MNI ​= ​Montreal neurological institute, OCD ​= ​obsessive compulsive disorder, PD ​= ​panic disorder, QoL ​= ​quality of life, SCH ​= ​subclinical hypothyroidism, SD ​= ​standard deviation, SF-36 ​= ​short form 36 questionnaire, Thy-PRO ​= ​thyroid-specific patient reported outcome, VAS ​= ​visual analogue scale.

**Table 3:** Study and patients characteristics of population-based studies.

ARTICLE	RESEARCH QUESTION	STUDY DESIGN	PATIENTS CHARACTERISTICS			
			Population	Sample size (N)	Mean Age in yrs (±SD, range) or range	Gender F (N)
**PATIENTS FROM THE GENERAL POPULATION**						
KRYSIAK 2016^**32**^	Is the association between hypothyroidism and sexuality a consequence of a hypometabolic state or thyroid autoimmunity, and is sexual dysfunction associated with mood disturbances?	Cross-sectional	General	68	30	68 (100%)
DELITALA 2016^**30**^	Is there an association between depressive symptoms and thyroid autoimmunity, determined by the presence of TPO-abs?	Cross-sectional	General population	3138	36.3-64.7	1763 (56%)
FJAELLEGAARD 2015 ^**31**^	What is the significance of elevated anti-TPO as a marker of poor well-being and depression in euthyroid individuals and individuals with SCH?	Cross-sectional	General population	7634	Median: 53.0 (43-63)	3938 (52%)
ISEME 2015 ^**34**^	What is the association between the presence of autoantibodies at baseline and change in depressive symptom score over 5 years follow-up?	Cohort study, retrospective	General population	1207 out of 2049	Median: 65.69 (±12.65, 55-85) (2049 participants)	965 (47%)
ITTERMANN 2015 ^**35**^	What is the association between TPO-abs and depression and anxiety?	Cross-sectional	General population	1644	Median: 50 (39-61)	776 (47.2%)
VAN DE VEN 2012 ^**36**^	Is there a relationship between the presence of TPO-abs and fatigue in euthyroid subjects?	Cross-sectional	General population	5439 out of 5897	55.6 (±17.9, 18-98) (5897 participants)	3101 (53%) (out of 5897 participants)
VAN DE VEN 2012 ^**37**^	What is the association between the presence of TPO-abs and the prevalence and severity of depression?	Cross-sectional	General population	1125	56.8 (±5.7)	546 (49%)
GRIGOROVA 2012 ^**38**^	What is the relationship between Tg-abs and performance on neuropsychological tests in healthy, euthyroid women?	Cross-sectional	General population	122	51 (±15.2, 25-75)	122 (100%)
ENGUM 2005 ^**39**^	What is the relationship between thyroid autoimmunity and depression or anxiety in a population-based sample?	Cross-sectional	General population	30175 (anti-TPO measured in 2445)	40-84	1737 (71%) (out of 2445 anti-TPO measured participants)
GRABE 2005 ^**40**^	Is autoimmune thyroiditis associated with mental and physical complaints in the general population?	Cross-sectional	General population	1006	>20	1006 (100%)
STRIEDER 2005 ^**41**^	Is there an association between TPO-abs, an early marker for AITD, and self-reported stress?	Cross-sectional	General population	759	18-65	759 (100%)
CARTA 2004 ^**33**^	What is the relationship between mood and anxiety disorders and thyroid autoimmunity?	Cross-sectional	General population	222	>18	127 (57.2%)
**PATIENTS FROM A PRIMARY CARE FACILITY**						
BUNEVICIUS 2007 ^**50**^	What is the impact of thyroid immunity, evident by hypo-echoic thyroid ultrasound pattern, on prevalence of depression and anxiety symptoms in a primary care setting?	Cross-sectional	Primary care	474	52.0 (18-89)	348 (73%)
KIRIM 2012 ^**51**^	Is the frequency of depression elevated in patients with chronic autoimmune thyroiditis and normal thyroid function?	Cross-sectional	Endocrinology Outpatient Clinic	201	38.0 (±11; 18-65)	197 (98%)
BAZZICHI 2007 ^**52**^	What are the characteristics of thyroid autoimmunity in patients affected by FM and what are the relationships between clinical data and symptoms?	Cross-sectional	Fibromyalgia	120	50.64 (±12.42, 18-75)	115 (96%)
**POSTPARTUM WOMEN**						
GROER 2013 ^**43**^	What is the relationship between TPO status, development of PPT and dysphoric moods across pregnancy and postpartum?	Cohort study, prospective	Post-partum women	135	≥18 and ≤45	135 (100%)
MCCOY 2008 ^**45**^	What is the relationship between quantified mood and thyroid measures?	Cohort study, prospective	Post-partum women	51	≥18	51 (100%)
HARRIS 1989 ^**46**^	What is the relationship between PPTD and thyroid antibodies, and mood disorders?	Cohort study, prospective	Post-partum women	147	17-40	147 (100%)
**PREGNANT WOMEN**						
WESSELOO 2018^**47**^	What is the association between a positive TPO-Ab status during early gestation and first-onset postpartum depression?	Cohort study, prospective	Pregnant women and post-partum	1075	30.4 (±3.5)	1075 (100%)
POP 2006 ^**48**^	What is the relation between thyroid parameters (TSH, FT4 and TPO-abs) and an episode of major depression at different trimesters during pregnancy?	Cohort study, prospective	Pregnant women	1017	29.0 (±0.5)	1017 (100%)
**PERIMENOPAUSAL WOMEN**						
POP 1998 ^**42**^	What is the relationship between autoimmune thyroid dysfunction and depression in perimenopausal women?	Cross-sectional	Perimenopausal women	583	49.9 (±2.2)	583 (100%)
**PATIENTS WITH ANOTHER AUTOIMMUNE DISEASE**						
AHMAD 2015 ^**44**^	How does AIT affect the clinical presentation of established RA with particular reference to FM and CWP?	Cohort study, retrospective	Patients with RA	204	58.23 (±13.06)	188 (92%)
CARTA 2002 ^**49**^	What is the relationship between celiac disease and psychiatric disorders and what is the relevance of associated thyroid disease in the development of psychiatric illnesses in celiac patients?	Case-control study, retrospective	Patients with coeliac disease	36	41.1 (±15.3, 18-64)	27 (75%)

AIT ​= ​autoimmune thyroiditis, AITD(s) ​= ​autoimmune thyroid disease(s), Anti-TPO ​= ​anti thyroid peroxidase, CWP ​= ​chronic widespread pain, F ​= ​female, FM ​= ​fibromyalgia, FT4 ​= ​free thyroxine 4, N ​= ​number, PPT ​= ​post-partum thyroiditis, PPTD ​= ​post-partum thyroid disease, RA ​= ​rheumatoid arthritis, SCH ​= ​subclinical hypothyroidism, SD ​= ​standard deviation, Tg-ab(s) ​= ​thyroglobulin antibody(-ies), TPO ​= ​thyroid peroxidase, TPO-abs(s) ​= ​thyroid peroxidase antibody(-ies), TSH ​= ​thyroid stimulating hormone.

**Table 4:** Results of population-based studies.

Article	Main outcome measure	Results			Authors’ conclusion	Do results support the hypothesis?
**PATIENTS FROM THE GENERAL POPULATION**						
KRYSIAK 2016 ^32^	Autoimmune SCH vs non-autoimmune SCH compared on multiple variables	Non-autoimmune SCH N = 17	Autoimmune SCH N = 17	P-value	Both autoimmune thyroiditis and subclinical hypothyroidism are associated with a lower total FSFI score, lower scores in selected FSFI domains and higher BDI-II score. These disturbances are particularly pronounced in women whose secondary hypothyroidism results from autoimmune thyroiditis. The obtained results suggest that both thyroid autoimmunity and thyroid hypofunction disturb female sexual function and that their deteriorating effect on women's sexuality are additive.	Yes
	BDI-II score (mean, SD)	11.3 (3.9)	15.6 (3.4)	< 0.05		
	Depressive symptoms (n, %)	6 (35)	10 (59)	< 0.05		
	Mild symptoms (n, %)	6 (35)	9 (53)	< 0.05		
	Moderate symptoms (n, %)	0 (0)	1 (6)	–		
	Severe symptoms (n, %)	0 (0)	0 (0)	–		
	FSFI score (mean, SD)	27.87 (3.62)	23.74 (4.00)	–		
	Sexual desire (mean, SD)	4.30 (0.48)	3.38 (0.51)	< 0.01		
	Sexual arousal (mean, SD)	4.75 (0.67)	4.25 (0.46)	–		
	Lubrication (mean, SD)	4.70 (0.51)	4.10 (0.48)	–		
	Orgasm (mean, SD)	4.38 (0.60)	3.95 (0.50)	–		
	Sexual satisfaction (mean, SD)	4.92 (0.68)	3.94 (0.47)	< 0.01		
	Dyspareunia	4.82 (0.65)	4.12 (0.60)	–		
DELITALA 2016 ^30^	Relation of TPO-abs and CES-D. Result of multiple regression analysis and logistic regression analysis, adjusted for age, sex, obesity (BMI≥30), smoking, and education.	CES-D Continuous:	Relation of TPO-abs + vs. TPO-abs –	β (se) −0.304 (0.394) P = 0.440	No support was found for an association between thyroid autoimmunity (TPO-abs) and depressive symptoms in a community-based cohort.	No
			Relation of TPO-abs titer	0.001 (0.001) P = 0.626		
		CES-D > 16:	Relation of TPO-abs + vs. TPO-abs –	OR (95% CI) 1.20 (0.75–2.60) P = 0.126		
			Relation of TPO-abs titer	1.00 (0.66–3.95) P = 0.300		
FJAELLEGAARD 2015 ^31^	Percentage of all euthyroid subjects with:	Anti-TPO –	Anti-TPO +	P-value	No significant differences were found in well-being or depression between euthyroid TPO-abs positive and TPO-abs negative individuals.	No
		N = 7015	N = 619			
	1. Depression assessed with MDI questionnaire on depression categories:					
	- 0–3% “No”	39	39	–		
	- 4–19% “Low”	56	56	–		
	- 20–25% “Medium”	3	2	0.8		
	- >25% “High”	2	3	–		
	- DSM-IV MDD	2	2	0.8		
	2. Well-being raw score ≥50%	85	86	0.4		
ISEME 2015 ^34^	Change in CES-D from baseline to follow-up (5yr)	TPO-abs –	TPO-abs +	Interaction term coefficient (*r*); 95% CI (P-value)	No significant association was found between change in CES-D score over time and TPO-abs.	No
	Baseline	4.47	3.95	–		
	5-year follow-up	6.85	6.56	–		
	Change	2.38	2.61	0.23; −1.28-1.75 (0.76)		
	Adjusted for variables (gender, cholesterol, hypertension, medication, smoking, BMI).					
	Baseline	4.16	3.06	–		
	5-year follow-up	6.41	5.42	–		
	Change	2.25	2.36	0.11; −2.23-2.45 (0.93)		
ITTERMANN 2015 ^35^	MDD, multivariable poisson regression models:		Anti-TPO-increased (M ≥ 60, F ≥ 100 IU/mL) N = 115 (7.0%)	P-value	This study detected significant associations between positive TPO-Abs and lifetime depression when excluding individuals with thyroid medication. Furthermore, no significant association was found between TPO-abs and recent depression in this study. A significant positive association between increased TPO-abs and MDD 12 months, but that finding was neither confirmed for positive TPO-abs nor for a BDI-II≥12.	Yes
			RR (95% Confidence Interval)			
	- Global		1.29 (0.78–2.11)	–		
	- Recurrent		1.18 (0.60–2.34)	–		
	- Last 12 months		2.88 (1.47–5.65)	–		
	- Global lifetime		–	–		
	- Global recurrent		–	–		
	- BDI-II ≥12		0.93 (0.52–1.65)	–		
	- Anxiety excl. specific phobias		1.80 (0.96–3.38)			
	MDD, multivariable poisson regression models:		Anti-TPO positive (≥200 IU/mL) N = 54 (3.3%) RR (95% Confidence Interval)			
	- Global		1.24 (0.54–2.84)	–		
	- Recurrent		1.47 (0.55–3.92)	–		
	- Last 12 months		1.78 (0.56–5.74)	–		
	- Global lifetime		2.14 (1.13–4.06)	0.020		
	- Global recurrent		3.30 (1.21–9.01)	0.020		
	- BDI-II ≥12		0.42 (0.13–1.34)	–		
	- Anxiety excl. specific phobias		1.89 (0.75–4.81)	–		
VAN DE VEN 2012 ^36^	Self-reported fatigue and scores of RAND-36 vitality subscale and SFQ in euthyroid subjects free of known thyroid disorder (N = 5439):	Euthyroid anti-TPO – N = 4870	Euthyroid anti-TPO + N = 569	RR or RC and [CI]	No association between the level of TPO-abs and fatigue was found.	No
	- Self reported fatigue	33.9%	34.6%	RR 1.0 [0.8–1.1]		
	- RAND-36 vitality subscale	66.3	66.3	RC 0.7 [-0.9-2.2]		
	- SFQ	11.1	11.4	RC 0.1 [-0.5-0.7]		
VAN DE VEN 2012 ^37^	Percentage of subjects with (number in brackets after % = number of patients that completed the specific questionnaire):	TPO-ab ≤12 kIU/l	TPO-ab >12 kIU/l	RR (95%CI), P-value	The presence of TPO-abs is associated with trait characteristics factors like neuroticism and lifetime diagnosis of depression, whereas thyroid function is not.	Yes
	- Current depression	15.3% (N = 791)	19.1% (N = 115)	1.2 (0.8–1.9)	The presence of TPO-abs may be a vulnerability marker for depression.	
	- Lifetime depression	16.7% (N = 882)	24.2% (N = 124)	1.4 (1.0–2.1), < 0.05		
	Scores:			Difference from reference group (95% CI), P-value	No significant relationship between the presence of TPO-abs and state markers of depression was found in the general population.	
	- BDI	5.1 (N = 791)	6.0 (N = 115)	0.74 (−0.2–1.7)		
	- EPQ-RSS neuroticism subscale	3.2 (N = 879)	4.1 (N = 121)	0.7 (0.1–1.3), < 0.05		
GRIGOROVA 2012 ^38^	Significant correlations between scores on the executive function tests and thyroid hormone levels: Design fluency perseverative errors (corrected) Design fluency (total errors) Word fluency (total errors) Trails B errors.	Higher Tg-ab levels were positively correlated with more errors on: - Trail Making Test Part B (r = 0.470; P = 0.000) - Word Fluency Test (r = 0.284; P = 0.023) - Design Fluency (r = 0.28; P = 0.045) test. The demographic, mood, and neuropsychological test data of all participants with Tg-Ab levels lower than 20mU/L were merged into a low (<20mU/L) Tg-ab group (N = 96) and compared to that of the women whose Tg-ab levels were >20mU/L (high Tg-ab group; N = 29). There were no significant differences between the groups on any of the demographic or mood scores. However, the women in the high Tg-ab group made significantly more perseverative errors on the Design Fluency test (P = 0.003) compared to women in the log Tg-ab group.			The hypothesis that higher levels of Tg-ab would be associated with worse performance on all of the neuropsychological tests was partially supported. Only on the Trails Making Test-Part B, the Design Fluency and the Word Fluency tests, higher levels of Tg-abs were associated with more errors. These findings suggest that higher levels of Tg-abs antibodies are related to poorer performance on tasks of executive functions.	Yes
ENGUM 2005 ^39^	Prevalence (%) in TPO-abs positive subjects (cut-off 200U/mL) compared to general population:	TPO-abs – (reference category): N = 29,180 (General population)	TPO-abs + N = 995	P-value	The presence of TPO-abs was not associated with depression or anxiety.	No
	- HADS-D (≥8)	13.2%	11.6%	0.125		
	- HADS-A (≥8)	16.7%	16.3%	0.709		
	Logistic regression analysis of depression or anxiety as dependent variables in relation to thyroid antibodies, when controlling for age, gender and thyroid function		OR adj. (95%)			
		1	0.92 (0.69–1.22) P = 0.557	HADS-D ≥8		
		1	0.76 (0.45–1.26) P = 0.285	HADS-D ≥11		
		1	0.93 (0.72–1.20) P = 0.584	HADS-A ≥8		
		1	1.18 (0.77–1.81) P = 0.447	HADS-A ≥11		
GRABE 2005 ^40^	Explorative comparison of symptoms between women, MANOVA (adjusted mean [SE]):	Euthyroid without goiter N = 961	Euthyroid Autoimmune thyroiditis N = 30	MANOVA	There is some preliminary evidence, that AIT, even without pathologic changes in thyroid hormones, could alter mental well-being at least in females. Therefore, AIT could be associated with negative well-being independently from the current thyroid function.	Yes
	- Tachycardia	1.6 [0.02]		*F* = 4.8; df = 1990; P = 0.03		
	- Anxiety	1.5 [0.02]		*F* = 7.1; df = 1990; P = 0.008		
	- Globus sensation	1.3 [0.02]		*F* = 1.7; df = 1990; P = 0.19		
	- Nausea	1.3 [0.02]		*F* = 2.5; df = 1, 990; P = 0.11		
	Adjusted for age, gender, education and marital status					
STRIEDER 2005 ^41^	Experienced stress in TPO-abs positive and TPO-abs negative euthyroid subjects. (Mean [SD])	TPO-abs – N = 576	TPO-abs + N = 183	P-value, observed [corrected for age]	No association between recently experienced stressful life events, daily hassles or mood and the presence or absence of TPO antibodies was found in euthyroid women.	No
	Recent life events - Total life events	11.2 [6.2]	10.3 [6.1]	0.09 [0.97]		
	- Unpleasant events	4.7 [3.4]	4.6 [3.5]	0.68 [0.68]		
	- Pleasant events	5.2 [3.5]	4.5 [3.5]	0.02 [0.66]		
	- Total unpleasantness	16.7 [12.4]	15.1 [11.0]	0.13 [0.68]		
	- Total pleasantness	18.9 [12.6]	15.9 [11.4]	0.01 [0.38]		
	Daily Hassles - Total number	25.2 [14.1]	23.8 [13.6]	0.24 [0.83]		
	- Intensity per hassle	1.3 [0.4]	1.3 [0.4]	0.52 [0.38]		
	- Total intensity of all	35.4 [25.5]	32.2 [22.9]	0.15 [0.57]		
	Positive and Negative affect schedule scale - Report negative feelings	22.2 [7.3]	22.1 [7.4]	0.89 [0.88]		
	- Report positive feelings	38.3 [5.3]	38.2 [5.1]	0.91 [0.91]		
CARTA 2004 ^33^	Association between anti-TPO+, mood and anxiety disorders:	OR anti-TPO + vs anti-TPO –		P-value (95% CI)	Anti-TPO positivity is associated with a higher lifetime risk of a diagnosis of one mood or anxiety disorder.	Yes
	- One anxiety diagnosis (GAD + PD + SP + ADNOS)	4.2		0.001 (1.9–38.8)		
	- One mood diagnosis (MDE + DD + DDNOS)	2.9		0.011 (1.4–6.6)		
	- GAD	2.7		0.058 (0.97–7.5)		
	- PD	5.4		0.096 (0.7–37.3)		
	- SP	3.6		0.111 (0.7–7.6)		
	- ADNOS	4.0		0.045 (1.1–15.5)		
	- MDE	2.7		0.033 (1.1–6.7)		
	- DD	5.2		0.250 (0.3–16.8)		
	- DDNOS	4.4		0.049 (1–19.3)		
**PATIENTS FROM A PRIMARY CARE FACILITY**						
BUNEVICIUS 2007 ^50^	Number of pre-menopausal women (N = 153) with:	Normo-echoic thyroid N = 137	Hypo-echoic thyroid (AITD) N = 16	P-value	Thyroid autoimmunity, evaluated by a relatively simple, cost effective but reliable technique, ultrasonographic imaging of the thyroid gland, is associated with mood symptoms in primary health care patients, especially in pre-menopausal women.	Yes
	- HADS depression >10	4 (3%)	3 (19%)	0.02		
	- HADS anxiety >10	27 (20%)	6 (38%)	0.2		
	- MINI diagnoses major depression	21 (15%)	3 (19%)	0.7		
	- MINI diagnoses AD:					
	Panic disorder	6 (4%)	2 (13%)	0.2		
	Social phobia	8 (6%)	2 (13%)	0.3		
	Generalized anxiety	30 (33%)	5 (31%)	0.4		
	- Depression or anxiety disorder	40 (29%)	8 (50%)	0.09		
KIRIM 2012 ^51^	Number of subjects HRDS levels positive for thyroid autoantibodies vs. negative.	Thyroid auto- antibodies – N = 107	Thyroid auto- antibodies + N = 94	P value	Patients with euthyroid chronic autoimmune thyroiditis showed an elevated frequency of depression and a higher rate of severe depression. HDRS scores were correlated to age only in the control group and not in patients with euthyroid chronic AIT, suggesting a possible link between depression and euthyroid Hashimoto's Disease.	Yes
	- Normal (0–7)	94 (87.9%)	8 (8.5%)	–		
	- Mild-medium (8-23)	13 (12.1%)	51 (54.3%)	–		
	- Severe-very severe (19–53)	0 (0%)	35 (37.2%)	–		
	Average HDRS value:	3.65 (±3.17)	16.05 (±6.05)	<0.001		
BAZZICHI 2007 ^52^	Percentage of FM patients with clinical characteristics:	Anti-TPO – N = 70	Anti-TPO + N = 50	P-value	The results suggest a relationship between thyroid autoimmunity and FM, and highlight the association between thyroid autoimmunity and some typical symptoms such as: dysuria, allodynia, sore throat, blurred vision and dry eyes. Thyroid autoimmunity is a marker of the severity of FM, especially if patients are in post-menopausal status.	Yes
	- Dry eyes	36.5%	56.0%	<0.05		
	- Burning/pain with urination	10.0%	36.0%	<0.01		
	- Allodynia	32.4%	73.5%	<0.01		
	- Blurred vision	22.5%	48.9%	<0.01		
	- Sore throat	16.9%	43.7%	<0.01		
**POSTPARTUM WOMEN**						
GROER 2013 ^43^	POMS-D and POMS-A at the time of pregnancy measurement.	Anti-TPO – N = 72	Anti-TPO + N = 63 (pregnant) N = 47 (post-partum)	The 63 TPO-positive pregnant women had statistically significantly higher scores on the POMS depression-dejection (POMS-D) subscale (8.5) compared to TPO-negative women (5.9) at the time of pregnancy measurement (P = 0.028). Depression symptom reports were higher postpartum for TPO-positive than PPT-negative mothers, F(1.129) = 9.1, P = 0.003. POMS-A subscale scores, F(1.131) = 6.4, P = 0.013, and total mood disturbance scores, F(1.130) = 5.3, P = 0.023, were also higher in the TPO-positive group than in the TPO-negative group.	In pregnant women, more clinical depression and higher depressive symptom scores were found when TPO positive, and the same pattern continued postpartum. The findings support a relationship between dysphoric moods and TPO antibody status across the peripartum period.	Yes
MCCOY 2008 ^45^	Score on 10-item EPDS score 4 weeks postpartum.	TPO-abs – N = 44	TPO-abs + N = 7 Seven subjects had positive antibody tests at 4 weeks postpartum.	P-value The 7 participants with positive antibody tests were more likely than their counterparts to have higher EPDS scores. P = 0.0428	TPO-abs + women tended to have higher scores on the EPDS at 4 weeks post-partum than TPO-abs – women, even when PPD was not present.	Yes
HARRIS 1989 ^46^	Psychiatric assessment according to DSM-III criteria for depressed mood by a psychiatrist on three questionnaires: The Rasking 3-area scale for depression, The MADS and The Edinburgh Postnatal depression scale.	Anti-TPO – N = 82 (56%)	Anti-TPO + N = 65 (44%) The frequency of cases of postnatal depression at the time of assessment was not significantly different in Ab + compared with Ab- women (x2 test). This was true for both microsomal and thyroglobulin antibody status.	P-value Not mentioned.	The presence of autoantibodies showed little association with depressed mood.	No
**PREGNANT WOMEN**						
WESSELOO 2018 ^47^	Risk of self-reported first-onset postpartum depression:	Anti-TPO + 121 (11.3%)	Anti-TPO – 954	Adjusted OR, P-value (95% CI)	Women with a positive TPO-ab status during early gestation are at increased risk for self-reported first-onset depression at four months postpartum, but not at other time points. This period coincides with the typical postpartum rebound phenomena of the maternal immune system, which suggest an overlap in the etiology of first-onset postpartum depression and auto-immune thyroid dysfunction.	Yes
	- 6 weeks	1.7%	2.1%	–		
	- 4 months	5.8% (7/121)	2.1%	3.8, 0.017 (1.3–11.6)∗		
	- 8 months	1.7%	3.0%	–		
	- 12 months	0.8%	2.2%	–		
POP 2006 ^48^	Assessment of depression by CIDI: Multiple logistic regression analysis in 1017 women at two different assessments during gestation		OR Increased TPO-abs titers (>35) N = 1017	95% CI	At 12 and 24 weeks gestation, an elevated titre of TPO-abs was significantly related to depression as well as other confounders.	Yes
	- 12 weeks gestation		2.1	1.1–5.8		
	- 24 weeks gestation		2.8	1.9–7.1		
**PERIMENOPAUSAL WOMAN**						
POP 1998 ^42^	Multiple logistic regression analysis with depression (score ≥12 on the Edinburgh Depression Scale) as dependent variable.	Low TPO-ab levels (<100 U/mL) N = 525 (90%)	High TPO-ab levels (≥100 U/mL) N = 58 (10%)	95% CI	Women with a high concentration of TPO-abs are at risk for depression, a relationship that still exists after adjustment for other (psycho-social) determinants of depression.	Yes
	OR	1	3.0	1.3–6.8		
		The occurrence of financial problems, caring for parents, a previous episode of depression in the woman's life, the occurrence of a major life event, and an elevated concentration of TPO-Ab (≥100 U/mL) were all significantly and independently related to depression.				
**PATIENTS WITH AUTOIMMUNE DISEASE**						
AHMAD 2015 ^44^		No Autoimmune Thyroid Disease N = 130	Autoimmune Thyroid Disease N = 74	P-value	This study shows a positive association between AIT and the presence of TPO-abs, and FM or CWP in patients with established RA.	Yes
	FM or CWP	17%	40%	<0.01		
		OR	OR (95%CI)			
	TPO-abs adjusted OR for FM (adjusted for age, sex, DM, BMI and spinal Degenerative Disc Disorder)	1	4.458 (1.950–10.191)	< 0.001		
CARTA 2002 ^49^		Anti-TPO – N = 25	Anti TPO+ N = 11	P-value	Celiac patients with positive anti-TPO were more frequently affected by lifetime MDD than celiac patients without anti-TPO. A similar significant association between PD and TPO + among celiac patients was found.	Yes
	Number of celiac patients effected with lifetime MDD	6 (24%)	9 (81.8%)	<0.01		
	Number of celiac patients effected with PD	1 (4%)	4 (36.4%)	<0.01		

ab = antibody, Adj. = adjusted, ADNOS = anxiety disorder not otherwise specified, AIT = autoimmune thyroiditis, AITD(s) = autoimmune thyroid disease(s), anti-TPO = anti thyroid peroxidase, anti-TPO+/- = anti-TPO positive/negative, BDI = Beck depression inventory, BMI = body mass index, CES-D = centre for epidemiological studies-depression scale, CI = confidence interval, CIDI = the composite international diagnostic interview, CWP = chronic widespread pain, DD = dysthymic disorder, DDNOS = depressive disorder not otherwise specified, df = degrees of freedom, DM = Diabetes Mellitus, DSM = diagnostic and statistical manual of mental disorders, EPDS = Edinburgh postnatal depression subscale, EPQ-RSS = Eysenck personality questionnaire revised short scale, F = female, FSFI = female sexual function index, GAD = generalized anxiety disorder, HADS = hospital anxiety and depression scale, HADS-A = HADS-anxiety, HADS-D = HADS-depression, HDRS = Hamilton depression rating scale, M = male, MADS = Montgomery-Asberg depression rating scale, MANOVA = multivariate analysis of variance, MDE = major depressive episode, MDI = WHO major (ICD-10) depression inventory, MDD = major depression diagnosis, MINI = mini international neuropsychiatric interview, N = number, OR = odds ratio, PD = panic disorder, POMS-A = profile of mood states checklists for anxiety, POMS-D = profile of mood states checklists for depression, PPD = post-partum depression, PPT = post-partum thyroiditis, RA = rheumatoid arthritis, RAND-36 = research and development-36, RC = regression coefficient, RR = relative risk, SCH = subclinical hypothyroidism, SD = standard deviation, SE = standard error, SFQ = shortened fatigue questionnaire, SP = social phobia, Tg-ab(s) = thyroglobulin antibody(-ies), TPO = thyroid peroxidase, TPO-ab(s) = thyroid peroxidase antibody(-ies), TPO-ab+/- = TPO-ab positive/negative, yr = year.

∗ Different sample: anti-TPO + (n = 119), anti-TPO–(n = 934). Self-reported first-onset depression rates 5.0% and 1.5% resp.

### 2.4 Statistical analysis

Due to different outcome measures and presentation of results in all studies, data could not be aggregated. Therefore, meta-analysis was not performed. Instead, a qualitative synthesis of the included data was performed.

## 3 Results

**Fig. 1:**
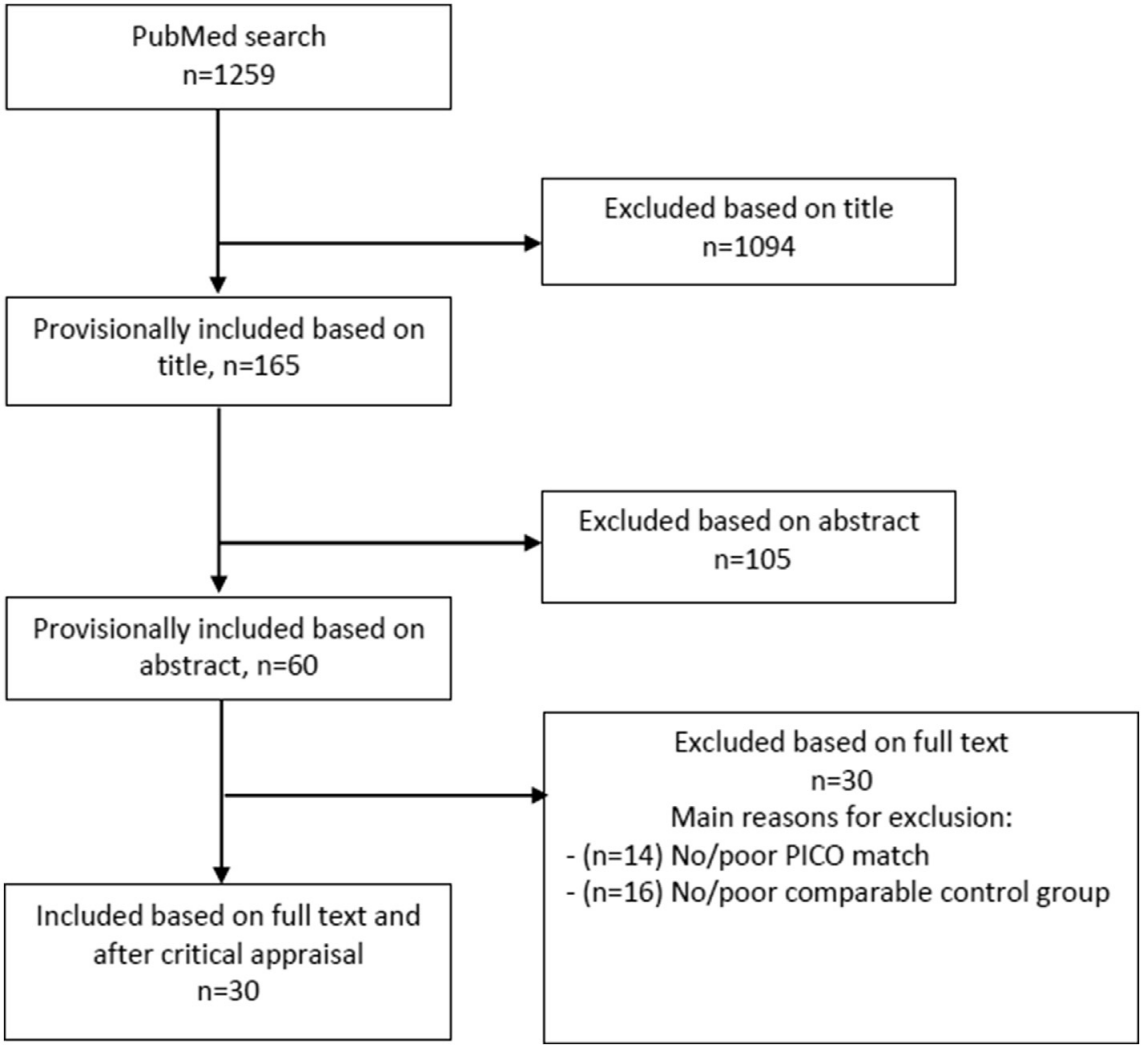
Flowchart illustrating the results of the literature search performed in this systematic review.

The PubMed search was performed on January 10th, 2020 in PubMed and yielded 1259 articles. We excluded 1229 articles through title, abstract or full text screening (n ​= ​1094, n ​= ​105, and n ​= ​30, respectively). A total of 30 articles was included in this systematic review (Fig. 1). Seven articles could be classified as disease-based studies, twenty-three as population-based studies. The results of the critical appraisal are shown in supplementary table 1 and the used PRISMA checklist is shown in supplementary table 2.

### 3.1 Study characteristics

For study characteristics see Table 1 and Table 3. In the disease-based studies sample size ranged from n ​= ​21 to n ​= ​379, and mean age from 35.1 to 54.6 years. In the population-based studies sample size ranged from n ​= ​36 to n ​= ​7,634, and mean age from 17 to 98 years. The included studies could be classified as cross-sectional (n ​= ​17), prospective cohort (n ​= ​8), retrospective cohort (n ​= ​4) or case-control (n ​= ​1).

### 3.2 Disease-based studies

The results of the individual included disease-based studies are summarized in Table 2. Five disease-based studies evaluated well-being in LT4 treated patients with HD compared with patients with non-autoimmune hypothyroidism, using the following questionnaires: DSM-IV mood and anxiety disorders, FIQ, FM comorbidity, General Symptom Questionnaire, MFI-20, QoL, SCID, ThyPRO, VAS (Table 4) [19,[28], [29], [30], [31]]. Three studies reported a significant relation between persisting symptoms and thyroid autoimmunity [19,30,31]. In two studies neurocognitive function was investigated with patient and control groups as described above, using the *d2 attention test.* [20,21] In one of these studies the results were also related to grey matter density of the left inferior frontal gyrus determined by brain magnetic resonance imaging [21]. Both studies showed a significant association between symptoms and thyroid autoimmunity.

Thyroid function was specified in six of the seven disease-based studies. TSH, free T4 and free T3 levels were similar in HD patients and controls (non-autoimmune hypothyroidism and euthyroid benign goitre patients) in four studies [[19], [20], [21],^31^]. In two studies TSH values were significantly lower in the control groups [28,30], that consisted of patients who underwent thyroidectomy because of differentiated thyroid cancer and who were subsequently treated with LT4 aiming at a suppressed TSH [30]. In one study all HD patients and controls were classified as euthyroid, but thyroid function was not specified [29].

Overall, five of the seven disease-based studies found a statistically significant association between persisting symptoms and the presence of thyroid autoimmunity.

### 3.3 Population-based studies

The results of the individual included population-based studies are summarized in Table 4. Large and representative samples of the general population were investigated in 12 of 23 population-based studies [[32], [33], [34], [35], [36], [37], [38], [39], [40], [41], [42], [43]]. Different tests and questionnaires were used to assess the presence of persisting symptoms (BDI, CES-D, EDS, Executive Function Test, EPQ-RSS, FSFI, HADS-D/-A, MDD, MDI, RAND-36, SFQ, Symptom checklist, Life Events Checklist). Krysiak et al., Ittermann et al. and van de Ven et al. used the Beck Depression Inventory (BDI), however data were presented differently [34,37,38]. The same applies to Delitala et al. and Iseme et al. concerning the Center for Epidemiological Studies Depression scale (CES-D) [32,36]. Seven out of these 12 general population studies showed a significant relation between symptoms and thyroid autoimmunity [34,35,37,38,40,42,44]. The other eleven studies were performed in populations from a primary care facility (n ​= ​3), postpartum women (n ​= ​3), pregnant women (n ​= ​2), perimenopausal women (n ​= ​1), and patients with another autoimmune disease (n ​= ​2) [[44], [45], [46], [47], [48], [49], [50], [51], [52], [53], [54]]. In these studies, the following tests and questionnaires were used to evaluate symptoms: CIDI, Clinical Characteristics FM, EPDS, HADS-D, HADS-A, HRDS, MADS, MINI, POMS-D, POMS-A, Prevalence of FM/CWP. A significant relation between symptoms and thyroid autoimmunity was described in the three studies of individuals from a primary care facility, two studies in postpartum women, in both studies of pregnant women, in the study of perimenopausal women, and in both studies of patients with another autoimmune disease [[45], [46], [47], [48], [49], [50], [51], [52], [53], [54]].

Overall, 16 of the 23 population-based studies reported a statistically significant association between symptoms and thyroid autoimmunity. However, the total number of people studied in the seven studies showing no association between symptoms and thyroid autoimmunity was much higher (n ​= ​20,769; study sample size range 147–7634) than the number of people in the 16 studies that did show an association (n ​= ​8038; study sample size range 36–1644).

## 4 Discussion

In this systematic review we have tried to answer the question whether or not the presence of thyroid autoimmunity is associated with persisting symptoms in HD patients. Twenty-one out of 30 well-designed studies included in the review (70%) reported a probable relation between the presence of thyroid autoimmunity, and (persisting) symptoms or lower QoL. Validity of the studies was evaluated through critical appraisal following the pillars of the NOS. The included studies were divided into studies evaluating LT4 treated patients with hypothyroidism due to HD versus patients with non-autoimmune hypothyroidism (disease-based studies), and (mostly healthy general) population-based studies. An association between the presence of thyroid autoimmunity and (persisting) symptoms was found in five of the seven disease-based studies, and in 16 of the 23 population-based studies. Due to great variety in tests, questionnaires and outcome measures among the studies, data could not be combined nor could a meta-analysis be performed. Yet, to our best knowledge this is the first systematic review on this topic.

In the population-based studies, most participants with and without markers of thyroid autoimmunity - mostly TPO-abs - had a normal thyroid function. Yet, in many of these studies a number of patients had (sub-clinical) hypothyroidism or hyperthyroidism. Although some studies reported a relation between thyroid function and symptoms [32,34,37,[46], [47], [48],^50^], most studies that reported an association between thyroid autoimmunity and symptoms did so after correction for thyroid function. In the disease-based studies, biochemically euthyroid auto-immune hypothyroid patients reported more symptoms than biochemically euthyroid non-autoimmune hypothyroid patients or euthyroid patients with a benign goitre. In most of these studies thyroid function was similar in the HD patients and controls. Although the factor suboptimal thyroid hormone treatment cannot be ruled out, we feel that ongoing (thyroid) autoimmunity may at least play an additional role in the persisting symptoms or lower QoL of these HD patients.

Thyroid autoimmunity as cause of persisting symptoms in treated HD patients has been suggested before. Leyhe et al. reported an association between cognitive and affective disorders, and (euthyroid) autoimmune thyroid disease in their review [55]. The well-designed study by Ott et al., included in this review, showed strong evidence for a relation between thyroid auto-immunity and persisting symptoms [19]. In this study, 426 consecutive euthyroid female patients who underwent thyroid surgery for benign thyroid disease (goitre) were evaluated. Removed thyroid glands were examined for lymphocytic infiltration, and based on histology results and pre-surgery anti-TPO levels an anti-TPO concentration cut-off for “true” inflammation (121.0 IU/mL) was calculated. Subsequently, anti-TPO negative and positive patients were compared with respect to pre-operatively symptoms and QoL. A significant association between anti-TPO levels and chronic fatigue, chronic irritability, chronic nervousness, and lower QoL levels was found.

Results of several other studies are also in support of the hypothesis that there is a relation between thyroid auto-immunity and (persisting) symptoms in euthyroid HD patients. In a cross-sectional study, Watt et al. evaluated health-related QoL, using the ThyPRO, in 199 patients with auto-immune hypothyroidism with TPO-abs levels >60IU/l [56]. No association between QoL scores and thyroid function tests was seen. However, in a multivariate model the TPO-abs level was related to goitre symptoms, depression and anxiety. Half of the studied patients were euthyroid at the time of the study and only 2% were overtly hypothyroid, which may explain that no relation between thyroid function test and QoL was found. Watt el al. concluded that the health-related QoL in patients with auto-immune hypothyroidism was related to TPO-abs level, but not thyroid function. A recent Norwegian randomized trial evaluated the effect of thyroidectomy versus no thyroid surgery on persisting symptoms in adequately LT4 treated HD patients. One hundred and fifty adult euthyroid HD patients with anti-TPO concentrations greater than 1000 IU/mL and persisting symptoms were studied. SF-36 general health score, fatigue score and chronic fatigue frequency improved only in the patients who underwent thyroidectomy [57]. The median serum TPO-abs concentration decreased sharply after thyroidectomy (2232–152 IU/mL). The combination of the decrease in anti-TPO concentrations and the observed clinical improvement suggests that thyroid autoimmunity plays a role in persisting symptoms in euthyroid HD patients. Yet, an important limitation of this study is that it was not blinded by performing a sham operation instead of “no thyroid surgery”. Therefore, a placebo effect of thyroid surgery cannot be ruled out. Because these two studies lacked control groups as defined in our methods, they were not included in this review.

With respect to the population-based studies included in this systematic review, two other studies showed a relation between presence of TPO-abs, and mood and anxiety disorders [35,58]. However, these two studies were not included in this review because the investigated populations consisted of psychiatric patients which were considered to be non-representative study groups. In all included general population studies - also in the seven studies that showed no significant difference between the groups with and without thyroid autoimmunity -, a proportion of the TPO-abs positive participants experienced symptoms, while another part did not. This fits within our hypothesis that thyroid autoantibodies and thus (low-grade) thyroiditis may be present and cause symptoms, even though thyroid function is not yet compromised.

A relation between the presence of thyroid autoimmunity and neurological or psychiatric symptoms has been recognized previously in SREAT, formerly known as “Hashimoto’s encephalopathy”. In this condition, affected persons have encephalopathy that was attributed to the presence of thyroid autoimmunity [59]. For a long time, TPO-abs crossing the blood-brain barrier were held responsible, but a causative role for these abs has never been proven. It is suggested that both thyroid and brain are targets of autoimmunity, hence the name SREAT, but how is still unknown [60]. SREAT is a rare condition with an estimated prevalence of 2.1/100,000 [61]. Yet, if there is a causal relation between the (thyroid) autoimmunity in persons with TPO-abs or patients with HD on the one hand, and (persisting) symptoms on the other hand - many symptoms may be traced back to (sensing by) the brain, and might be viewed as mild brain dysfunction.

In addition to SREAT or HD patients with (persisting) symptoms, brain autoimmunity may also play a role in the pathogenesis of (late) neurological symptoms in other disorders. For example, it was shown that herpes simplex virus encephalitis may promote the development of neuronal autoantibodies targeting mostly the NMDA receptor causing subsequent autoimmune encephalitis [62]. During the SARS-CoV-2 pandemic neurological symptoms in COVID-19 patients (disabling fatigue, anosmia, Guillain-Barré syndrome and encephalopathy) can persist or reemerge after clearance of SARS-CoV-2, and are hypothesized to be caused by cross-reactive antibodies generated in response to the primary viral infection [63]. These examples illustrate that a secondary brain autoimmunity may cause neurological symptoms, which is in line with our postulated hypothesis.

A strength of this systematic review is the long period from which articles are included. Although the scientific literature database was also searched for relevant articles published before 1980, none were found. The most important limitation of this systematic review is that we were not able to compare or aggregate results of studies due to different outcome measurements, and different cut-off values used for TPO-abs. For future research we therefore suggest uniformity in measurements. For example, within the field of rheumatology OMERACT (Outcome Measurements in Rheumatology) is an initiative to increase the standardization of outcomes, with successful results [64]. Since a meta-analysis could not be performed, we could also not correct for differences in sample size; the association between thyroid autoimmunity and symptoms that we found in the population-based studies may therefore be less pronounced, since the studies that showed no association were performed in considerably larger populations. Another limitation of the population-based studies is that thyroid function was not always specified, and that a possible relation between TSH or thyroid hormone levels, and symptoms was not always evaluated or corrected for. Therefore, in these studies minor differences in thyroid function may have contributed to symptoms that were attributed to thyroid auto-immunity alone. Furthermore, studies in populations with another diagnosed autoimmune disease (such as celiac disease and rheumatoid arthritis) are prone for selection bias due the chance of overt and latent poly-autoimmunity. In cases of (latent) poly-autoimmunity, it will be difficult to ascertain a possible relation between thyroid autoimmunity alone and persisting symptoms. However, the latencies for other autoantibodies may hypothetically also play a role in the clinical course of patients with HD. Therefore, it is interesting to evaluate the presence of other autoantibodies in relation to persisting symptoms of HD. Only two of the herein included studies measured other autoantibodies, but did not analyze any relationship between the autoantibodies [31,36]. Six studies reported diabetes and/or rheumatoid arthritis in their population characteristics [52], of which two studies stated that the presence of diabetes mellitus was not a confounder [19,46], and three adjusted their results for the presence of diabetes and/or rheumatoid arthritis [36,38,39]. Future studies are needed to evaluate an additional role of latencies for other autoantibodies in treated HD patients with persisting symptoms. Finally, the results reported in this review may be influenced by publication bias, although we think the chance of publication bias is small as studies both in favour and against our hypothesis were published and subsequently included in this review.

### 4.1 Conclusions

In summary, the majority of the included studies in this systematic review reported an association between thyroid autoimmunity and persisting symptoms or low QoL in patients with HD. Meta-analysis of data was not possible due to the wide variety of used outcome measures. Several possible causes of persisting symptoms in hypothyroid patients have been proposed previously, like LT4 not being the optimal drug for treatment of hypothyroidism and *DIO2* gene polymorphisms. However, given the overall results of this systematic review (thyroid) autoimmunity per se may also play a role in persisting symptoms in a part of HD patients. This however, needs further investigation as the found association does not prove a causality. Conducting a randomized placebo-controlled trial, evaluating the effect of additional immunomodulating treatment, for example intravenous immunoglobulins versus placebo, may further elucidate the role of thyroid autoimmunity in persisting symptoms in patients with HD.

## Registration and protocols

This review was not registered and no protocol was not prepared.

## Declaration of competing interest

The authors declare that they have no known competing financial interests or personal relationships that could have appeared to influence the work reported in this paper.
