# Supplementary material for: Persisting symptoms in patients with Hashimoto’s disease despite normal thyroid hormone levels: Does thyroid autoimmunity play a role? A systematic review
Source: J Transl Autoimmun. 2021 Apr 15;4:100101. doi: 10.1016/j.jtauto.2021.100101 (PMC8122172; doi:10.1016/j.jtauto.2021.100101)
Supplement: Multimedia component 1 [file mmc1.docx]

***Supplementary table 1******.*** *Critical appraisal of the 30 included articles by an eight-item checklist according Newcastle-Ottawa Scale (NOS), Quality Assessment Form for Cohort studies^‡^.*

|  | Representiveness of exposed cohort | Selection of the non-exposed cohort | Ascertainment of exposure | Demonstration outcome was not present at start study | Comparability cohorts | Assessment of outcome | Follow-up length | Follow-up adequacy |
| --- | --- | --- | --- | --- | --- | --- | --- | --- |
|  | **Selection** | | | | **Comparison** | **Outcome** | | |
| POPULATION BASED STUDIES | | | | | | | | |
| 2018 Wesseloo | 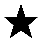 | 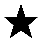 | 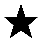 | 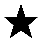 | 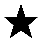^**^ | C | 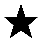 | 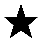 |
| 2016 Krysiak | C | 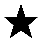 | 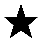 | 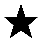 | 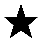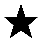 | C | - | - |
| 2016 Delitala | 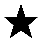 | 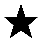 | 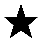 | 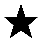 | 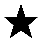^*^ | C | - | - |
| 2015 Ahmad | 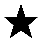 | 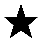 | 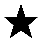 | B | 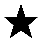^*^ | 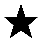 | - | - |
| 2015 Fjaellegaard | 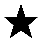 | 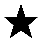 | 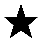 | B | 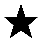 | C | - | - |
| 2015 Iseme | 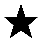 | 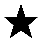 | 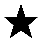 | 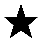 | 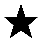^*^ | C | 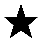 | 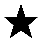 |
| 2015 Ittermann | 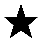 | 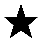 | 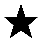 | 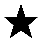 | 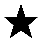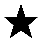 | 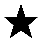 | - | - |
| 2015 Zivaljevic | 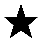 | 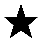 | 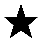 | 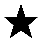 | 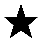 | 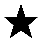 | 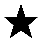 | 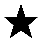 |
| 2014 Giynas Ayhan | 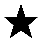 | 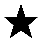 | 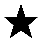 | B | 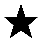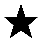 | E | - | - |
| 2013 Groer | 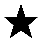 | 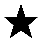 | 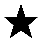 | B | 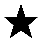 | C | 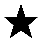 | 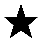 |
| 2013 Leyhe | 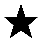 | 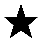 | 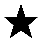 | 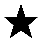 | 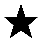^*^ | 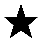 | - | - |
| 2012 Kirim | C | 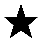 | 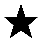 | B | C | D | - | - |
| 2012 van de Ven | 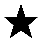 | 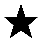 | 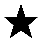 | B | 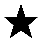^*^ | C | - | - |
| 2012 van de Ven | 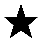 | 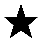 | 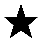 | B | 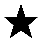^*^ | C | - | - |
| 2012 Bazzichi | 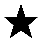 | 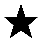 | 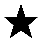 | B | 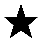 | C/D | - | - |
| 2012 Grigorova | 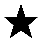 | 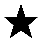 | 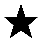 | 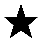 | 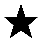 | 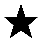 | - | - |
| 2012 Louwerens | 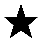 | B | 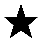 | B | 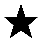^*^ | C | - | - |
| 2011 Ott | 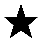 | 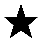 | 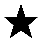 | B | 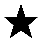^**^ | C | - | - |
| 2008 Leyhe | 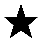 | 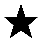 | 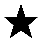 | 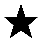 | 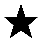^*^ | 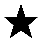 | - | - |
| 2008 McCoy | 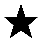 | 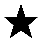 |  |  |  | C | ^†^ | C |
| 2007 Bazzichi |  |  |  |  | - | C | - | - |
| 2007 Bunevicius |  |  |  | B | ** |  | - | - |
| 2006 Pop |  |  |  | B |  |  |  |  |
| 2005 Engum |  |  |  | B | ^*^ | C | - | - |
| 2005 Grabe |  |  |  |  |  | C | - | - |
| 2005 Strieder |  |  |  |  | ^**^ | C | - | - |
| 2004 Carta |  |  |  | B | * | D | - | - |
| 2002 Carta |  | B |  | B | C | C | - | - |
| 1998 Pop |  |  |  | B | - | C | - | - |
| 1989 Harris |  |  |  | B | ** |  | B |  |

* Corrected for age and gender, but not for marital status.

** Corrected for age, but not for gender and marital status.

† Follow-up period shorter than other studies.

‡ Follow-up period could not be measured in the included cross-sectional studies, therefore the thresholds to AHRQ-standards are not applicable in those studies.

Thresholds for converting the Newcastle-Ottawa scales to AHRQ standards (good, fair, and poor):

**Good quality:** 3 or 4 stars in selection domain AND 1 or 2 stars in comparability domain AND 2 or 3 stars in outcome/exposure domain

**Fair quality:** 2 stars in selection domain AND 1 or 2 stars in comparability domain AND 2 or 3 stars in outcome/exposure domain

**Poor quality:** 0 or 1 star in selection domain OR 0 stars in comparability domain OR 0 or 1 stars in outcome/exposure domain
